# Investigating Benzoic Acid Derivatives as Potential Atomic Layer Deposition Inhibitors Using Nanoscale Infrared Spectroscopy

**DOI:** 10.3390/nano15030164

**Published:** 2025-01-22

**Authors:** Saumya Satyarthy, Mark Cheng, Ayanjeet Ghosh

**Affiliations:** 1 Department of Chemistry and Biochemistry, The University of Alabama, Tuscaloosa, AL 35487, USA; ssatyarthy@crimson.ua.edu; 2 Department of Electrical and Computer Engineering, The University of Alabama, Tuscaloosa, AL 35487, USA; mmcheng@eng.ua.edu

**Keywords:** atomic layer deposition, self-assembled monolayers, atomic force microscopy-infrared spectroscopy, carboxylic acid inhibitors, zinc oxide deposition, surface chemistry and coordination

## Abstract

Area-selective atomic layer deposition (AS-ALD) is a technique utilized for the fabrication of patterned thin films in the semiconductor industry due to its capability to produce uniform and conformal structures with control over thickness at the atomic scale level. In AS-ALD, surfaces are functionalized such that only specific locations exhibit ALD growth, thus leading to spatial selectivity. Self-assembled monolayers (SAMs) are commonly used as ALD inhibiting agents for AS-ALD. However, the choice of organic molecules as viable options for AS-ALD remains limited and the precise effects of ALD nucleation and exposure to ALD conditions on the structure of SAMs is yet to be fully understood. In this work, we investigate the potential of small molecule carboxylates as ALD inhibitors, namely benzoic acid and two of its derivatives, 4-trifluoromethyl benzoic acid (TBA), and 3,5-Bis (trifluoromethyl)benzoic acid (BTBA) and demonstrate that monolayers of all three molecules are viable options for applications in ALD blocking. We find that the fluorinated SAMs are better ALD inhibitors; however, this property arises not from the hydrophobicity but the coordination chemistry of the SAM. Using nanoscale infrared spectroscopy, we probe the buried monolayer interface to demonstrate that the distribution of carboxylate coordination states and their evolution is correlated with ALD growth, highlighting the importance of the interfacial chemistry in optimizing and assessing ALD inhibitors.

## 1. Introduction

**Figure 1:**
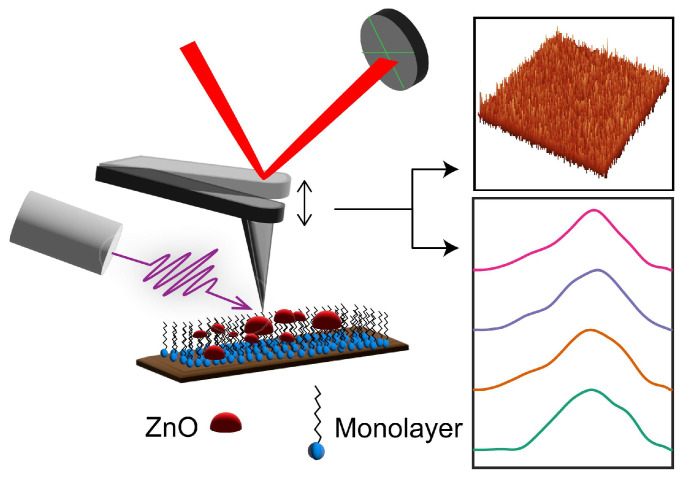
AFM-IR schematic. A pulsed tunable laser is focused on a sample near the AFM tip. The cantilever oscillations induced by photothermal expansion of the sample resulting from IR absorption can be leveraged to calculate the absorption cross section, thus providing a localized nanoscale IR spectrum. The pink, purple, orange, and green lines in the figure represent the IR spectra collected from the different locations in the corresponding AFM image.

The fabrication of nanoelectronics in the semiconductor industry predominantly utilizes top-down processing techniques, including deposition, photolithography, and etching [1,2,3,4,5]. With advances in device miniaturization, these conventional methodologies are encountering substantial limitations in pattern resolution and alignment precision, underscoring the imperative need for the development of innovative process technologies to address these challenges. Atomic layer deposition (ALD) is a widely used technique in semiconductor fabrication to deposit thin films, owing to its unparalleled capability of atomic-level control over the film’s thickness and composition. ALD’s capability to deposit high-quality films at low temperatures has enabled the development of passivation and barrier layers that enhance device stability and efficiency. The ability of ALD to produce uniform, high-quality thin films on complex 3D structures makes it an invaluable tool for scaling down devices and developing advanced architectures like gate-all-around (GAA) and channel-all-around (CAA) transistors. By providing in-situ composition control and vertical gradient engineering, ALD facilitates the fine-tuning of electrical properties in multi-component oxide semiconductors [6,7,8,9,10,11,12,13,14,15]. By employing self-limiting chemical reactions, ALD allows for uniform coatings on complex three-dimensional structures, making it indispensable for advanced electronic and nanotechnology applications [16,17]. Area-selective ALD (AS-ALD), an extension of the ALD process, is an additive nanomanufacturing technique wherein ALD nucleation is initiated only on specific surface sites of a predetermined chemistry while avoiding all other types of surface sites [18,19,20,21]. This selective nucleation enables further spatial control of the film composition beyond the capabilities of conventional ALD, which is vital for scaling of semiconductors to 5 nm and below [22,23,24]. The basic premise of AS-ALD involves patterning substrates such that specific areas are chemically inert to ALD reactants and block/resist ALD growth while other untreated areas allow ALD growth as normal [19]. This blocking can be achieved via chemisorption and self-assembly of monolayers of molecules with inert tail groups. The appeal of this approach lies in the fact that it does not necessitate the design of special ALD precursors [7,25,26,27,28,29,30,31,32,33,34]. The self-assembled monolayers (SAMs) of ALD inhibitors feature three essential components: a reactive headgroup for selective surface binding, a tail group that renders the film inert to ALD chemistry, and a backbone that ensures the formation of a densely packed monolayer through van der Waals dispersion forces [35,36,37,38,39,40,41,42]. SAMs inhibit ALD by blocking the diffusion of ALD precursors to the substrate surface and by replacing the active sites that would otherwise react with the ALD precursors, thereby preventing nucleation and growth. However, this approach has been limited by the lack of suitable inhibitors, with organo-silanes and organo-phosphonic acids being the main class of molecules that have been demonstrated as viable ALD blockers. Therefore, there is a pressing need for exploring and assessing alternative classes of inhibitors. Small molecule inhibitors (SMIs) for AS-ALD have recently attracted attention as alternatives to SAMs [43,44]. SMIs offer some key attributes, such as vapor-phase delivery, smaller size, etc., that can allow for better precision and scaling for high-volume manufacturing [45]. Recent studies by Bent and coworkers have demonstrated the potential of such an SMI, namely methyl-sulfonic-acid, as ALD-blocking agents. The potential of carboxylates as ALD inhibitors has also gained attention and has been investigated in recent work. Carboxylates can enable new AS-ALD routes that are not viable with silanes and phosphonic acids, owing to their wide range of tail chemistries and diverse selective adsorption on different oxides and crystal facets. However, the main challenge with developing new classes of ALD inhibitors, such as carboxylates, is the lack of understanding of the fundamental mechanisms underlying the selectivity of surface reactions. Inhibitor layers can degrade or desorb and lose their blocking capability with increasing ALD cycles, but the underlying mechanism remains largely unstudied. Morphological defects and packing inefficiencies in SAMs can also contribute to localized ALD nucleation [17,43,46,47,48,49,50]. However, direct spectroscopic and morphological measurements of SAMs before and after ALD have been scarce, leaving questions about the extent and nature of SAM degradation and its correlation with ALD growth unanswered [51,52,53,54,55]. This is largely due to limited capabilities in analyzing the chemistry of SAMs at the nanoscale before and after ALD cycles. X-ray photoelectron spectroscopy (XPS) and infrared (IR) spectroscopy are two techniques commonly employed in conjunction with ALD. However, neither of these techniques can reliably probe the SAM after ALD growth. Furthermore, these techniques offer spatially averaged insights, which can be skewed by the presence of localized variations in SAM chemistry. These limitations can be addressed by AFM-IR, which can simultaneously characterize the heterogeneity of monolayer chemistry and surface morphology with a nanoscale spatial resolution by integrating atomic force microscopy (AFM) with IR spectroscopy. A key strength of AFM-IR is the ability to probe the buried SAM under the ALD-deposited metal oxide films, which is enabled by its indirect determination of the IR cross section through the photothermal response of the sample (Figure 1). This also allows for avoiding scattering artifacts that may plague conventional spectroscopic measurements. A key advantage of integrating AFM with IR spectroscopy is that spectra can be correlated with morphological variations and interpreted accordingly, which is not possible from conventional spectroscopy, where any spectral variation arising from morphological aberrations cannot be delineated from the fundamental characteristics of the specimen. The capabilities of AFM-IR have been recently extended to characterizing buried interfaces and specifically towards mapping SAMs after ALD [56].

In this work, we investigate the efficacy of small carboxylates as effective inhibitors for ZnO ALD by employing SAMs of benzoic acid (BA), 4-(trifluoromethyl)benzoic acid (TBA), and 3,5-bis (trifluoromethyl)benzoic acid (BTBA) on cobalt (Co) substrates. Cobalt is a key material in semiconductor technology, particularly in the fabrication of interconnects. Copper has been extensively used for interconnects in semiconductors; however, diffusion of copper atoms can lead to reliability issues. As a result, alternatives such as cobalt, ruthenium, and nickel have been explored as viable materials for interconnects [57,58]. Furthermore, cobalt also acts as an excellent barrier layer in copper interconnects, preventing diffusion and consequent circuit failure [59,60]. Therefore, the choice of cobalt substrates for this study is extremely relevant to current fabrication standards for interconnects. Using cobalt substrates, we have systematically assessed the three inhibitors to gain fundamental insights into the molecular origins of their ALD-blocking abilities. Our results reveal that both TBA and BTBA exhibit selective ALD inhibition compared to both BA and untreated substrates and block ALD growth for up to 25 cycles. Importantly, this observed loss of ALD selectivity does not result from the degradation of the SAMs, as evidenced by AFM-IR spectra. However, we do observe a significant difference in the coordination between the carboxylate headgroups of TBA and BTBA with the metal substrate and their evolution with ALD compared to BA, which suggests that the coordination mode of the carboxylate headgroups plays a more important role in ALD inhibition compared to the expected differences in monolayer packing and Lewis basicities of the carboxylate moiety. The stability and effectiveness of these small molecules in preventing ALD growth underscore their potential in enhancing the precision and efficiency of nanomanufacturing processes. Our results can pave the way for the application of these molecules for ALD inhibition and AS-ALD on different substrates in the future.

## 2. Materials and Methods

### 2.1. Materials

A 4-(Trifluoromethyl) benzoic acid (≥97%, Sigma-Aldrich, St. Louis, MO, USA) and 3,5-Bis (trifluoromethyl) benzoic acid (≥97%, Sigma-Aldrich) were used as surface passivation molecules. Ethanol (200 proof, ≥99.5%, Sigma-Aldrich) was used as the solvent for dissolving both the chemicals. Prime-grade silicon (Si) wafers (Platypus Technologies, Fitchburg, WI, USA) were used as a supporting substrate for the metal layer coating. ALD precursors were diethylzinc (DEZ) (98%, Strem Chemicals, Newburyport, MA, USA) and deionized water (DI H_2_O). The carrier and purging gas for the ALD process was ultra-high purity nitrogen (99.999%, Airgas, Radnor, PA, USA).

### 2.2. Sample Preparation

Cobalt (Co) films with a thickness of 400 nm were fabricated using a DC magnetron sputtering system (AJA International Inc., Scituate, MA, USA), operated in ultra-high vacuum conditions. The deposition process utilized an off-axis configuration on silicon substrates pre-coated with a 10 nm aluminum adhesion layer. The sputtering was performed in an argon atmosphere at a pressure of 5 mTorr, with the base pressure of the system maintained at 5 × 10^−9^ Torr. Substrate temperature was held steady at 100 °C throughout the deposition. Uniformity was ensured by rotating the substrate at 30 rpm, and the growth rate was precisely monitored using a quartz crystal microbalance (QCM). Before monolayer fabrication, the Co substrates underwent a cleaning process consisting of three ethanol washes, followed by drying with a nitrogen stream. Subsequently, the surfaces were treated with UV ozone for one minute using an Ossila ozone cleaner to ensure optimal cleanliness. The substrates were then stored under vacuum conditions to prevent air-induced oxidation. For monolayer formation, 2 mM solutions of benzoic acid, 4-(trifluoromethyl) benzoic acid, and 3,5-bis (trifluoromethyl) benzoic acid were prepared in ethanol. Co substrates were fully immersed in these solutions for 24 h, after which they were rinsed thoroughly with ethanol and dried under a nitrogen flow. This preparation step ensured the substrates were ready for subsequent atomic layer deposition (ALD) experiments.

### 2.3. Atomic Layer Deposition on Monolayer Substrates

A flat-bottom ceramic boat was utilized as the sample holder during the ALD process. Diethylzinc (DEZ) served as the ZnO precursor, while deionized (DI) water was used as the counter-reactant. The ALD reactions were conducted at a temperature of 100 °C. Each ALD cycle included the following sequence: a 400 s initial N_2_ pre-purge, a 1 s DEZ pulse causing a temporary pressure increase of approximately 0.2 Torr, followed by a 15-s N_2_ purge, a 1 s DI water dose also inducing a similar transient pressure rise, and a final 45 s N_2_ purge. The overall reaction was carried out at a pressure of roughly 1 Torr, with a nitrogen flow rate of 250 sccm.

### 2.4. Characterization of Monolayers

X-ray photoelectron spectroscopy (XPS) was utilized to analyze surface chemistry and quantify both self-assembled monolayers (SAMs) and atomic layer deposition (ALD) growth. Measurements were performed with a VersaProbe II instrument, which employs a monochromatic Al Kα X-ray source (1486.6 eV) operated at 24.5 W, with a pass energy of 187.85 eV and a detection limit of 0.1%. The experiments were conducted at the Advanced Materials Characterization Facility, University of Alabama-Birmingham. Data processing and analysis were carried out using the Casa XPS software (version 2.3.25PR1.0). Atomic force microscopy-infrared (AFM-IR) measurements were conducted using a Bruker NanoIR3 system, integrating atomic force microscopy with a quantum cascade infrared laser (MIRCAT, Daylight Solutions). The imaging was carried out in contact mode at room temperature, with the ambient humidity controlled to approximately 5% through dry air purging. AFM-IR images were acquired at a resolution of 500 × 500 pixels. AFM image analysis was conducted using Gwyddion software (version 2.62), while the IR spectra were collected with a resolution of 2 cm^−1^. Spectral denoising was performed using a (2,5) Savitzky–Golay filter and a three-point moving average filter. To minimize variability in tip-sample interactions, all spectra were normalized to their maximum intensity. MATLAB (version R2023a) was employed for further data analysis and visualization.

## 3. Results and Discussion

**Figure 2:**
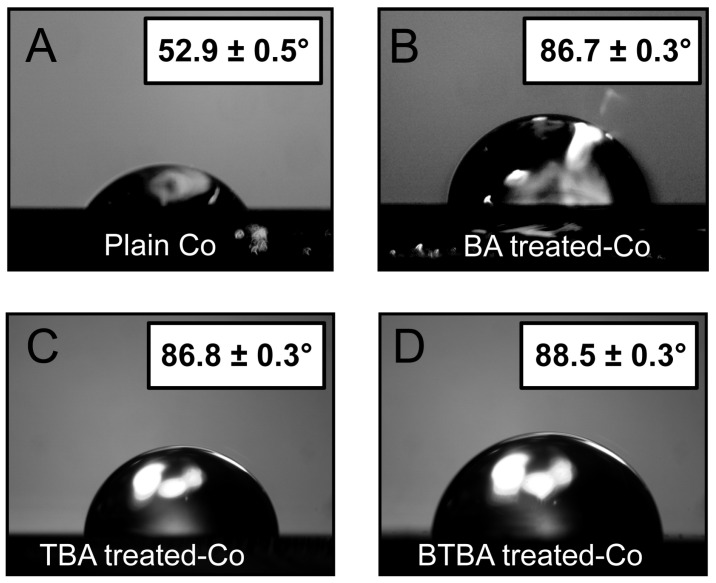
Water contact angle (WCA) images: (**A**) plain Co; (**B**) BA on Co; (**C**) TBA on Co; and (**D**) BTBA on Co.

Self-assembled monolayers (SAMs) were generated by immersing the Co substrates in an ethanolic solution of BA, TBA, and BTBA for 24 h. To verify if the incubation led to the formation of monolayers, we employed water contact angle (WCA) measurements, which reflect the hydrophilicity of the sample surface. Densely packed carboxylic acid SAMs with hydrophobic benzene groups at the surface are expected to greatly reduce the hydrophilicity and hence increase the contact angle of water. The WCA for plain cobalt was found to be 52.9 ± 0.5°, indicating its relatively low hydrophobicity. A similar observation was reported in the literature for cobalt oxide (Co_3_O_4_) [61]. However, upon incubation in BA, the contact angle increased to 86.7 ± 0.3°, a value consistent with surface modification studies involving BA on other substrates [62], suggesting an increase in hydrophobicity of the surface, which in turn indicates monolayer formation. Almost similar hydrophobicity was observed with the addition of fluorinated benzoic acids. Specifically, the contact angle increased to 86.8 ± 0.3° with the addition of 4-trifluoromethyl benzoic acid and to 88.5 ± 0.3° with the addition of 3,5-bistrifluoromethyl benzoic acid. These findings are consistent with previous reports that have demonstrated the role of fluorine atoms in enhancing surface hydrophobicity [63,64,65,66]. These results (Figure 2) indicate that monolayer growth of the small benzoic acid derivatives significantly improves the hydrophobicity of cobalt surfaces. We note that all the SAMs have similar hydrophobicity, leading to the expectation of similar ALD-blocking abilities for all of them. Furthermore, the observed WCA values do not reflect a highly hydrophobic surface, such as those observed for phosphonic acid monolayers. This can arise from the surface roughness and poor packing of the SAMs and would suggest less efficient ALD-blocking ability for these SAMs compared to phosphonic acid. We discuss the implications of this finding in detail later.

**Figure 3:**
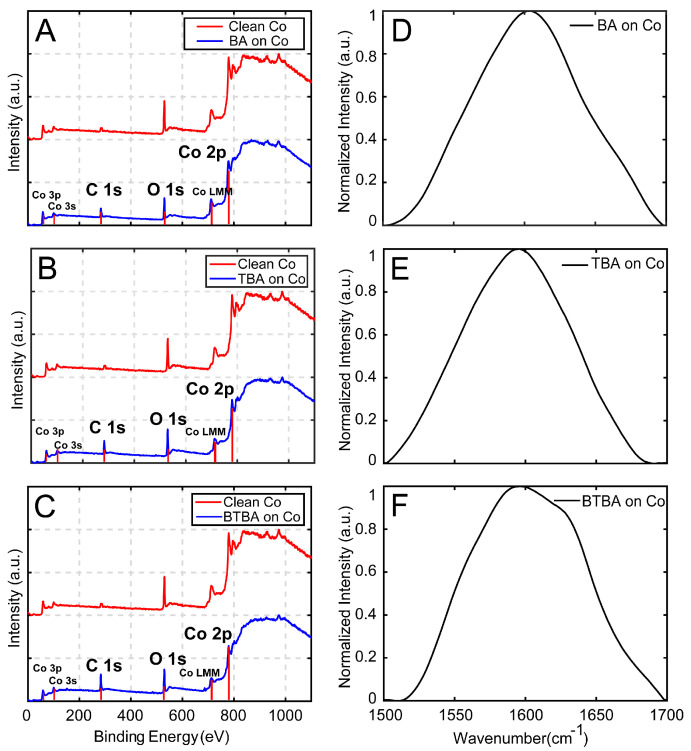
Comparison of XPS spectra of bare and SAM treated on cobalt (**A**) BA on cobalt (**B**) TBA on cobalt, and (**C**) BTBA on cobalt. AFM-IR spectra (**D**–**F**) represent the average infrared spectra for BA, TBA, and BTBA SAMs on cobalt, respectively, in the 1500–1700 cm^−1^ range. The broad peaks centered around ~1580 cm^−1^ corresponds to the asymmetric stretching of the carboxylate headgroup, confirming monolayer formation.

To further verify the formation of SAMs, we conducted X-ray photoelectron spectroscopy (XPS) and AFM-IR measurements. As shown in Figure 3A–C, the C1s peak intensity at 284.5 eV increased for BA-, TBA-, and BTBA-treated Co substrates, indicating the successful formation of monolayers. Additionally, the O 1s and Co 2p peaks are clearly visible in the XPS spectra, confirming the preservation of cobalt’s chemical states. AFM-IR experiments were conducted on a 5 µm × 5 µm area on the treated substrates. The average spectra recorded for each substrate in the spectral range of 1500-1700 cm^−1^ are shown in Figure 3D–F. The IR spectra of all the substrates exhibit a broad peak in this spectral region centered at approximately 1580 cm^−1^, corresponding to the asymmetric stretching mode of the carboxylate headgroup of the benzoic acid moieties [67,68,69,70]. The presence of this band thus further confirms the formation of monolayers. The spectra exhibit notable differences between BA, TBA, and BTBA, particularly in terms of the linewidth and presence of shoulders, underlying substructure variations due to different coordination modes; we discuss the structural implications of the spectra in detail later in the manuscript. Collectively, the XPS and AFM-IR spectra conclusively demonstrate the formation of monolayers that can be employed as ALD-blocking agents.

**Figure 4:**
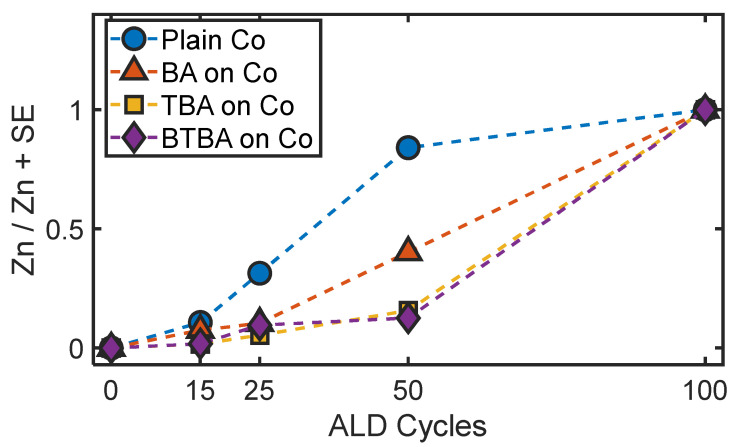
Zn/(Zn + surface element) values after different ALD cycles obtained from XPS compositional analysis for plain Co, BA-treated Co, TBA-treated Co, and BTBA-treated Co.

ZnO ALD was performed on the SAM-treated and clean Co substrates for varying ALD cycles (0, 15, 25, 50, and 100) to assess the blocking ability of the SAMs and the influence of ALD conditions and nucleation on them. ALD growth on the substrates was tracked by calculating the ratio of Zn/Zn + SE, where Zn and SE represent the abundance of Zn and the non-SAM surface element (SE) (i.e., Co), as determined from XPS compositional analysis. This ratio, denoted as θ, essentially corresponds to the atomic abundance of Zn relative to the substrate metal SE after varying numbers of ALD cycles. The value of θ can range from 0 to 1; the former represents a surface with no ZnO deposition, while the latter reflects a scenario where ALD nucleation has led to the formation of a film of ZnO thick enough that the electrons from the surface element signal cannot be detected. This typically corresponds to a film thickness of ~10 nm. Figure 4 shows the comparison of the θ values, as determined from XPS, on bare and SAMs-treated Co substrates after 0, 15, 25, 50, and 100 cycles of ZnO ALD cycles. On BA-treated Co, nucleation also occurs after 15 cycles, whereas TBA- and BTBA-treated cobalt nucleation starts after 25 cycles with a lower θ value indicating an inhibitory effect. In contrast, for bare Co, nucleation occurs within 15 cycles, with significantly higher ZnO deposition compared to the treated substrates, which is reflected in the sharp rise in the θ value. This is consistent with our previous studies on stearic acid monolayers [56]. TBA- and BTBA-treated Co inhibit significant ZnO growth compared to untreated cobalt for up to 50 cycles, but at 100 cycles, a dense ZnO layer is formed. In comparison, BA-treated Co is less effective at blocking ALD, as indicated by larger θ values. These results indicate that TBA and BTBA molecules can be viable alternatives to phosphonic or carboxylate SAMs that are typically used for ALD inhibition on metal substrates such as Co [71,72]. BA, on the other hand, exhibits somewhat lesser efficiency at inhibiting ALD growth. It should be noted that the focus of our work is not identifying the best set of parameters that lead to maximal ALD inhibition by SAMs but rather demonstrating the relative ALD-blocking capabilities under a given set of experimental conditions. Hence, it is possible that the sample fabrication parameters can be tuned to improve the blocking ability of all the SAMs observed here. Nonetheless, the above results confirm that TBA and BTBA SAMs can be used as potential ALD-blocking agents.

**Figure 5:**
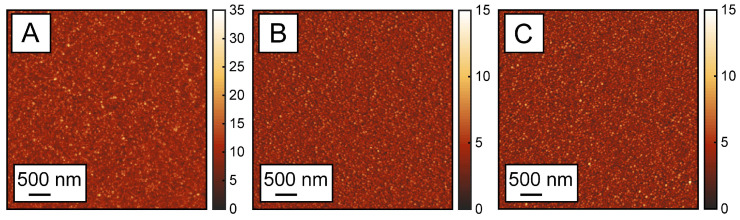
AFM topographs of (**A**) BA, (**B**) TBA, and (**C**) BTBA on cobalt before ALD. Each image represents a 5 µm by 5 µm area.

These observations also raise important questions regarding the origins of the different ALD-blocking abilities of the three SAMs. Inhibition of ALD nucleation is a complex function of multiple parameters, such as monolayer packing, surface quality/roughness, and substrate fabrication conditions. Since the same conditions were used for the fabrication of all the monolayers, the differences in ALD blocking between BA, TBA, and BTBA can be attributed to a different factor. The water contact angle measurements indicate very similar hydrophobicities for all the SAMs, suggesting that the observed ALD-blocking properties do not likely arise from variations in surface hydrophobicities between the SAMs. In terms of packing efficiency, TBA and BTBA are anticipated to exhibit lower packing efficiency than benzoic acid (BA) due to steric hindrance introduced by additional CF_3_ substituents. Consequently, if ALD growth were governed solely by packing efficiency, BA would exhibit better blocking in comparison to TBA and BTBA. However, as demonstrated in Figure 4, we observe an opposite trend from the XPS measurements. Taken together, these findings suggest that the chemistry of the SAM, rather than packing efficiency and hydrophobicity, underpins ALD growth characteristics. Another factor that can affect ALD growth is the surface morphology of the SAMs. To verify if the observed differences can be attributed to variation in surface roughness, we performed AFM measurements and calculated the surface roughness of the SAMs on each substrate (Figure 5A–C) The RMS roughness of the BA SAM surface on Co was determined to be 1.7 nm, while the TBA SAMs on Co had a roughness of 0.983 nm, and the BTBA SAMs had a roughness of 0.893 nm. The height profiles extracted from the AFM images further illustrate this variation in the surface topography of the SAM-treated cobalt substrates. The corresponding AFM images and height profiles are provided in the Supporting Information (Figure S4). While this trend in surface roughness correlates with the differences in ALD growth times, since the variations are small, it is likely that additional factors, such as monolayer chemistry, also contribute to the observed differences in the ALD blocking efficiency.

**Figure 6:**
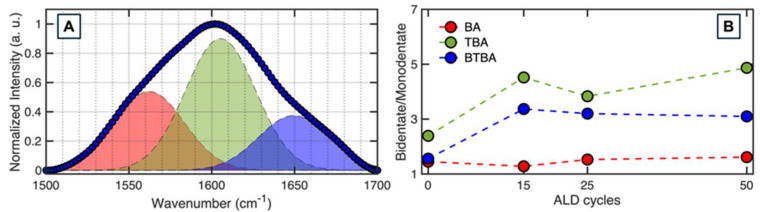
(**A**) Representative spectral deconvolution of AFM−IR spectra of BA on Co, with the spectra optimally fit to three Gaussian bands, each representing distinct coordination states of the carboxylate group. (**B**) Relative evolution of bidentate and monodentate coordination populations on BA, TBA, and BTBA with varying ALD cycles, illustrating the relationship between monolayer chemistry and ALD growth progression.

To elucidate the role of the interfacial chemistry on ALD growth, we used AFM-IR to acquire spectra of the SAMs after different ALD cycles. A broad, asymmetric spectral band was observed for the carboxylate vibration for all the SAMs, which can be further deconvoluted into three distinct components. Figure 6A shows the spectral fits for AFM-IR spectra of BA on cobalt after 0 ALD cycles. Other spectra and corresponding fits are shown in the Supporting Information. The carboxylate vibrational mode was evident in AFM-IR spectra at all times, ruling out any significant desorption of the SAM, which can also be a potential factor behind ALD nucleation. We hence focused on the evolution of the spectra with ALD cycles to determine the role of monolayer chemistry in ZnO deposition. Carboxylate groups are known to bond to metals through either monodentate or bidentate coordination. The bidentate carboxylate binding modes are known to exhibit lower vibrational frequencies compared to monodentate species. Additionally, there can be electrostatic or ionic interactions between the physisorbed carboxylate moiety and the metal site, without forming a bond, leading to vibrational frequencies that are typically intermediate to bidentate and monodentate states [73,74,75,76]. Furthermore, when considering the molecular origins of the different spectral sub-bands as seen in Figure 6A, it is important to note that benzene and its derivatives have a characteristic mode at ~1600 cm^−1^ arising from the phenyl ring vibrations [77,78]. Hence, the lowest frequency band observed herein can be assigned to bidentate coordination, while the highest frequency band can be attributed to monodentate binding. The peak at ~1600 cm^−1^ likely arises from the phenyl ring, although a potential contribution from a sub-population of ionic, physisorbed species cannot be ruled out. The interfacial monolayer chemistry at any point during ALD is reflected in the relative populations of the above coordination states. Therefore, to determine if there are any significant differences in the distribution of coordination states between the three SAMs, we calculated the areas under the curve (AUCs) for each band, which represents the effective population or concentration of each spectral component and subsequently determined the relative bidentate–monodentate distribution. The results, shown in Figure 6B, reveal a key difference between the SAMs that is consistent with the observed trends in ALD growth. We observe that the relative population of bidentate to monodentate states shifts with successive ALD cycles, showing markedly different trends between BA and TBA/BTBA (Figure 6B). For BA, the coordination distribution remains relatively unchanged, exhibiting a small increase after 25 cycles, whereas TBA and BTBA exhibit a significantly more pronounced increase in the proportion of bidentate states as ALD progresses. These findings corroborate previous research on stearic acid SAMs [56], where analogous structural evolution was observed. In organic SAMs, such as octadecylphosphonic acid, the coordination state distribution is critical for monolayer structural integrity [79,80,81]. Our results indicate that carboxylate coordination chemistry plays an equally important role in small molecule carboxylates, influencing blocking efficacy. It is also possible that the coordination distribution also compensates for the putative lack of efficient packing arising from the steric effect of the CF_3_ substituents. Overall, these findings provide new insights into the structural evolution of carboxylate SAMs during ALD and its implications for their blocking capabilities. In addition, the above results also provide a chemical and spectral basis for assessing ALD blocking of carboxylate SAMs, wherein a spectral measurement of the SAMs and subsequent analysis of binding modes can be reflective of ALD growth.

## 4. Conclusions

In summary, we demonstrated in this work the viability of small molecule carboxylates as viable ALD inhibitors for applications in AS-ALD. Specifically, we showed that monolayers of benzoic acid and two of its CF_3_-substituted derivatives can block ZnO ALD growth to varying degrees using XPS spectroscopy. We focused on a single substrate, cobalt, while investigating the ALD blocking efficiency of three different inhibitors of varying functionalities. This approach allows us to systematically evaluate the influence of molecular functionalization on inhibition performance and to develop a robust understanding of the molecular design principles before extending to other substrates. Interestingly, we did not find any significant variations in surface morphology and hydrophobicities of the SAMs, as indicated by water contact angle and AFM measurements, suggesting that the interfacial chemistry and structural organization of the monolayer are important parameters that affect ALD growth. Using AFM-IR, we probed the buried monolayer interface with increasing ALD growth and showed that the distribution of coordination states of the carboxylate headgroup and its evolution is significantly different between the SAMs. The two benzoic acid derivatives that exhibit better ALD inhibition both exhibit an increase in the relative abundance of bidentate coordination states with ALD cycles. In contrast, benzoic acid has a lower bidentate to monodentate ratio, which also remains relatively unchanged with ALD. Taken together, these findings highlight a pivotal role of the monolayer chemistry underlying ALD nucleation and growth beyond the conventional morphological and packing parameters. The insights gained from this study emphasize the importance of optimizing the monolayer chemistry for achieving better ALD selectivity and pave the way for the design of more effective ALD inhibitors. This contributes to advancements in surface modification technologies, enhancing the control and precision of thin-film deposition in various applications.

## Data Availability

All the data required to reach the conclusions made in this report have been provided in the manuscript and Supporting Information. Additional data are available upon reasonable request.

## Supplementary Materials

The following supporting information can be downloaded at https://www.mdpi.com/article/10.3390/nano15030164/s1: Figure S1: AFM-IR spectra of BA monolayers after different ALD cycles; Figure S2: AFM-IR spectra of TBA monolayers after different ALD cycles; Figure S3: AFM-IR spectra of BTBA monolayers after different ALD cycles; Figure S4: Height profiles extracted from AFM images of (A) BA on cobalt, (B) TBA on cobalt, and (C) BTBA on cobalt surfaces. The height profiles (D) were extracted along the dashed lines in A–C.
