# Supplementary material for: Investigating Benzoic Acid Derivatives as Potential Atomic Layer Deposition Inhibitors Using Nanoscale Infrared Spectroscopy
Source: Nanomaterials (Basel). 2025 Jan 22;15(3):164. doi: 10.3390/nano15030164 (PMC11821111; doi:10.3390/nano15030164)
Supplement: Supplementary file 1 [file nanomaterials-15-00164-s001.zip › nanomaterials-3381753-supplementary.pdf]

Supporting Information

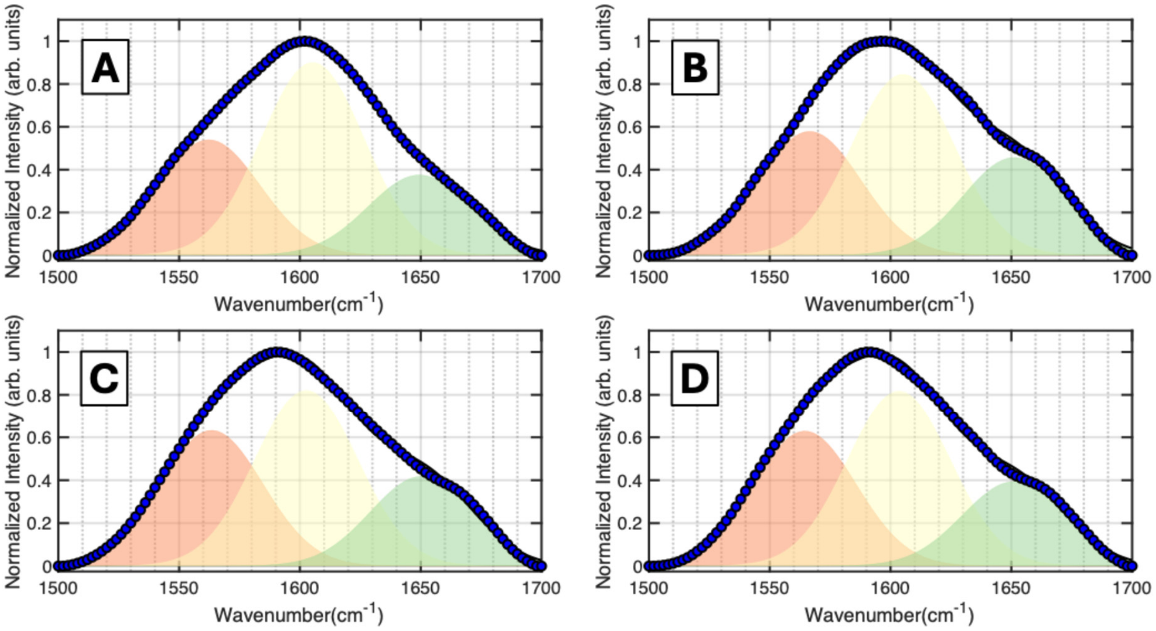

Figure S1. Normalized AFM-IR spectra corresponding to the 1500-1700-cm^{-1} region and corresponding spectral fits for benzoic acid (BA) after (A) 0, (B) 15, (C) 25 and (D) 50 ALD cycles.

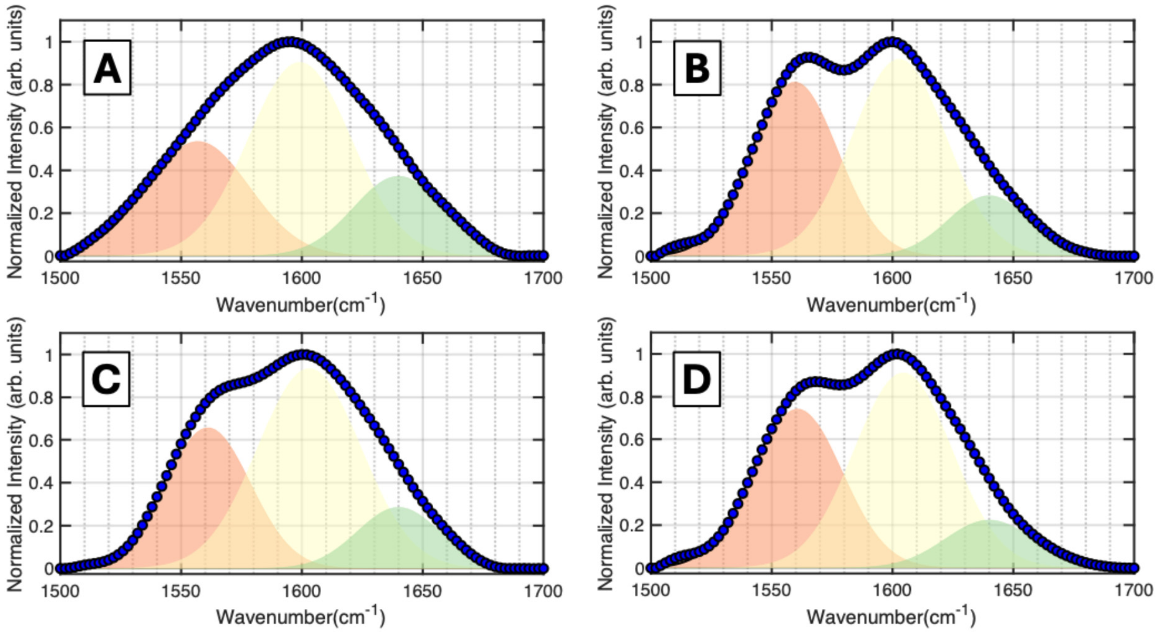

Figure S2. Normalized AFM-IR spectra corresponding to the 1500-1700-cm^{-1} region and corresponding spectral fits for 4-trifluoromethyl benzoic acid (TBA) after (A) 0, (B) 15, (C) 25 and (D) 50 ALD cycles.

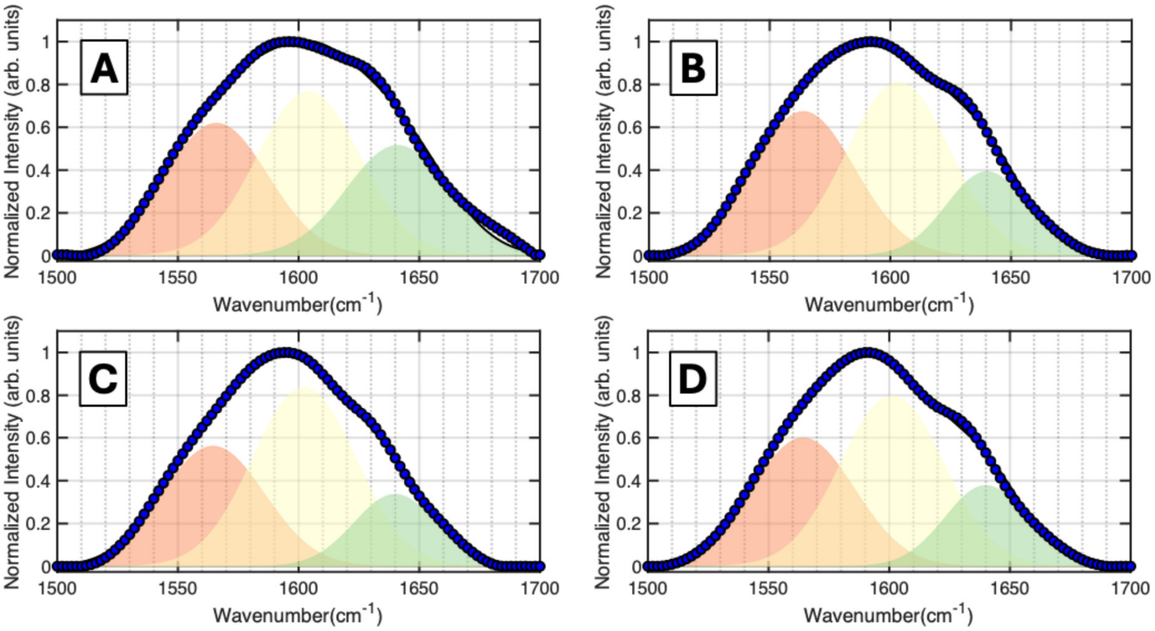

Figure S3. Normalized AFM-IR spectra corresponding to the 1500-1700-cm^{-1} region and corresponding spectral fits for 3,5-Bis(trifluoromethyl)benzoic acid (BTBA) after (A) 0, (B) 15, (C) 25 and (D) 50 ALD cycles.

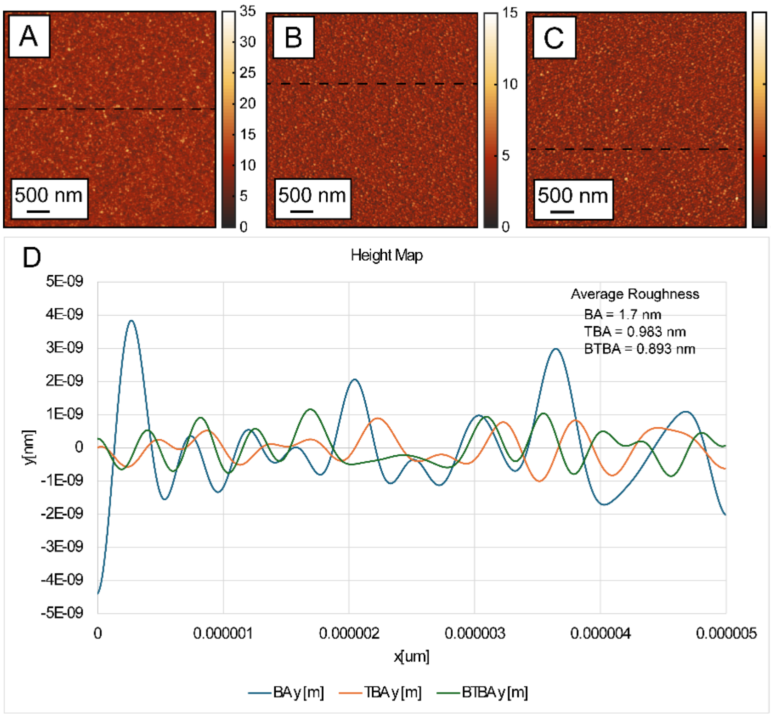

Figure S4: Height profiles extracted from AFM images of (A) BA on cobalt, (B) TBA on cobalt, and (C) BTBA on cobalt surfaces. The height profiles (D) were extracted along the dashed lines in A-C.
